# Phonological Assessment Instrument: evidence of content validity and response processes

**DOI:** 10.1590/2317-1782/20232022324en

**Published:** 2023-10-23

**Authors:** Ana Carolina Sartori Bernardi, Camila Botura, Giovana Sopezack Alves, Letícia Pacheco Ribas

**Affiliations:** 1 Universidade de Ciências da Saúde de Porto Alegre - UFCSPA - Porto Alegre (RS), Brasil.; 2 Departamento de Fonoaudiologia, Universidade de Ciências da Saúde de Porto Alegre - UFCSPA - Porto Alegre (RS), Brasil.

**Keywords:** Validation Study, Reproducibility of Results, Speech, Child, Language Tests, Speech Sound Disorder, Speech, Language and Hearing Sciences

## Abstract

**Purpose:**

to demonstrate the validity of content and the validity of response processes of an instrument intended for the phonological assessment of children.

**Methods:**

validation was carried out in two stages by two different groups of judges, a group of specialists and a group of non-specialists. The first group, composed of three expert judges, evaluated the 123 lexical items after creating the instrument, judging the applicability of the figures in the context of child assessment, and suggesting adjustments to compose the content. From the observations, the instrument was adapted and directed to the group of non-specialist judges who, through the application of the instrument, had their responses evaluated according to the ease or difficulty of eliciting the instrument's items.

**Results:**

The predictions obtained positive results for content validity and response processes.

**Conclusion:**

the study allowed to improve the test items more judiciously, benefiting clinical and scientific use.

## INTRODUCTION

Language is configured as an inherently human ability capable of objectively representing abstract thought through a complex system of shared codes^(1)^. Working at the service of interpersonal communication, language is composed of linguistic domains that contemplate use, form, and content^(2)^. Phonology is responsible for the functional study of phonemes, which are the minimum sound units capable of establishing the distinction between words of the same language^(2)^.

Gradually, language is acquired and developed through a hierarchy that includes all language domains. In the case of acquiring mastery of phonology, the child must organize the different sounds that make up the phonological system of their mother tongue so that it stabilizes. Studies show that the typical acquisition of language occurs up to 5 years of age^(1,3)^, considering the complexity of the distinctive features of phonemes^(3)^.

However, when there is no adequate development of phonology, speech production errors are observed, as is the case of Phonological Disorder (PD)^^1^^ ^(3)^. This is characterized^(4)^ in children over 4 years of age, who mostly present consonant exchanges in speech. In addition, having auditory thresholds within normal standards, not demonstrating alterations in the lexicon and syntax concerning expressive language, absence of evident neurological alterations, as well as normal cognitive abilities and intact comprehension ability. Therefore, it is necessary to carry out hearing tests, language assessments, and verification of the child's intelligence, to guarantee the correct diagnosis of PD.

To better understand this picture, in addition to carrying out all the assessments that confirm the diagnosis, it is necessary to carry out an effective phonological assessment, since only this can describe in detail the changes in the individual's speech^(5)^. It is important, therefore, to have an efficient method that assesses the child's phonological inventory, seeking a parameter of how their development and speech are progressing. The systematization of the evaluation will allow the accurate comparison of the child's phonological system with that of the target language, allowing the detailed investigation of the aspects that compose speech^(6)^. This entire diagnostic process must consist of tests with valid, reliable, and accurate interpretations^(7)^ so that the diagnosis is as appropriate as possible. To be valid, the assessment instrument must gather evidence that it measures what it purports to measure. To be reliable, the test needs to indicate whether it is reproducible over time and whether there is control over measurement errors^(8,9)^.

Therefore, the instrument must go through validity stages to collect evidence, consisting of validity of content, response processes, and construct. The validity of content and response processes are those that arise during the constitution of the test. The content assessment takes place right after the theoretical elaboration of an instrument and is carried out with the help of one or more groups of specialists willing to judge, independently, each item that makes up the test prototype. Next, the process-response evaluates part of the target audience; it is important to understand what are the greatest difficulties and facilities found by the subjects during the evaluation so that the test can be improved. The construct, on the other hand, consists of analyzing the instrument in terms of a representative sample of a domain^(8,10)^.

In Brazil, few speech-language instruments standardize their methodological pathways for the elaboration of valid and reliable tests^(11)^. The lack of validation of phonological assessment protocols in Brazilian Portuguese (BP) may impair the safety of clinical evidence to draw an accurate diagnosis, adequate conduct, and correct intervention planning. Although there are already tests available to aid in the evaluation and diagnosis, the most used instruments^(12)^, such as the Child's Phonological Assessment (AFC)^(13)^ and the ABFW - Child Language Test - Phonology^(14)^ have limitations and have not yet been have scientifically proven psychometric indicators of validity and reliability^(11)^. There are others, such as the Phonological Assessment Instrument (INFONO)^(12)^ and the Speech Assessment Instrument for Acoustic Analysis (IAFAC)^(15)^, but less publicized and not yet available for clinical use. Therefore, it is extremely important to carry out a validation study to bring advances in the area, seeking a scientifically proven gold standard for the evaluation of the phonological domain in BP. This may help in the diagnostic process, in addition to providing parameters for several studies. The present study aims to analyze the evidence of the content stages and response processes in the validity of the Phonological Assessment Instrument (IAF)^^2^^ , which is already used by some speech therapists.

## METHODS

This study was approved by the Research Ethics Committee (CEP) of a federal university under number 5.045.533. The research study corresponds to an observational, controlled cross-sectional, descriptive, and quantitative study, whose data were used for the content validity and response processes of the IAF.

The IAF is a software designed to evaluate the child's speech sound system efficiently, thoroughly, and optimally. The instrument was elaborated with 123 words, belonging to children's vocabulary, extracted from popular children's stories, easily represented in an image or photo, and of the noun type, with an image corresponding to each lexical item. The items were carefully selected so that the words included all consonant phonemes in all syllabic positions in BP, with five occurrences of each phoneme and syllable position, totaling 235 phonemic possibilities. The collection of the child's speech should occur from the naming of each of the images, by observing the illustrations or photographs, which takes approximately 10 minutes for the application. The evaluator must record the audio of the speech collection, and later listen to and observe the children's elicitations and register the information to the software. This process takes between 10 and 30 minutes, depending on the evaluator's practice and skill. After entering the data into the instrument, the results are automatically generated and expressed in descriptive and quantitative reports by: degree of speech severity, contrastive analysis, phonological processes, and change in distinctive features.

### Content validity

After the theoretical construction of the instrument, three expert judges were invited to judge the 123 items in the prototype. The judges who signed the Free and Informed Consent Form (ICF) and who were minimally masters in linguistics with expertise in typical and atypical phonological acquisition participated in the research. They should indicate the level of adequacy of each lexical item for the proposal and choose, between two options, the image that best represents it. At this stage, they should answer the question “Is the target word adequate to belong in a child speech assessment instrument?” quantitatively, through an electronic form, organized on a Likert scale^(16)^ for each item numbered from 1 to 4, as explained in Chart 1.

**Chart 1:** Likert scale for content classification

N	Correspondence
1	Item not suitable for a child speech assessment instrument
2	Item needs revision to be suitable for a child speech assessment instrument.
3	Item suitable for a child speech assessment instrument, although it needs minor changes.
4	Absolutely suitable item for a child speech assessment instrument.

For the qualitative approach, experts should justify their choices with their own criteria, recorded in descriptive comments on the same electronic form. For answers 1 or 2, the judges were instructed to suggest at least one new word for replacement, considering the same aspects listed during the elaboration of the instrument (that is, contemplating all consonant phonemes in all syllabic positions of BP). To understand the level of intra and inter-judge correspondence, the Content Validity Index (CVI) was calculated.

### Validity of response processes

After feedback from the expert judges, the necessary sample size was calculated to determine a Kappa coefficient of 0.80, significantly higher than 0.60, indicating good agreement and with an estimated 25% prevalence of PD ^(17)^. For the significance of 5% and power of 80%, the result was a minimum sample of 165 children.

The IAF was applied to a group of students, aged between 5 years and 8 years and 11 months, from a public school in the municipal network of the city of Porto Alegre. The sample of this study is composed of data from 176 children, with typical phonological acquisition or with PD, considering that none has auditory, neurological, and/or cognitive alterations, school difficulties, history of neuropsychomotor delay, and/or intercurrences in pregnancy or childbirth. This was checked through prior assessment and information collected in interviews with those responsible. All parents or guardians signed the ICF and Authorization for Audio Use; and, in the case of children over 7 years old, they also signed a Term of Assent.

Based on the sample's speech collections, a fourth judge, independent and blinded, classified the subjects' answers according to the level of difficulty observed in each of the lexical items. The answers were labeled according to the need for intervention by the applicator, described in Chart 2. From this, the internal consistency was calculated for the total items of the IAF by Cronbach's Alpha and by the percentage of recognition of each of the items by the frequency production of the target word.

**Chart 2:** Likert scale for use in response processes

N	Correspondence
0	Spontaneous nomination.
1	Needed tips and/or close method.
2	Required delayed imitation.
3	Required direct imitation.

## RESULTS

### Content validity

The analysis of the three expert judges regarding the prototype of the IAF instrument indicated a CVI of 0.98, which represents a very good index for the content validity of the test. To compose the instrument, those images in which at least two of the three judges agreed were chosen.

As can be seen in Table 1, the vast majority of lexical items showed maximum adequacy, represented by the code “4” on the Likert scale, to belong to a child speech assessment instrument. The judges classified the words according to their own criteria, namely: the word is or is not frequent in children's universe; whether or not the word is good for target verification; and whether the word provides adequate spontaneous naming.

**Table 1:** Content validity index (CVI) of the instrument according to each judge

Judges	CVI
A	1.0
B	0.98
C	0.96
Total	**0.98**
Judges’ polarization	**0.95**

**Caption:** CVI = Content Validity Index

The criteria described by the judges were similar, despite having been defined individually and independently in an essay text box on the form used, which explains the percentage obtained in the calculation of the CVI and the homogeneity of the answers, in which 116 items reached the maximum convergence. However, seven of the items presented divergences, they were: “bucket/balde”, “bicycle/bicicleta”, “gum/chiclete”, “iron/ferro”, “snow/neve”, “drone/zangão” and “zombie/zumbi”.

In these cases, only one of the three judges considered that the target words were not adequate, which was represented by the proper CVI of 0.75. For most items, there was no suggestion of a new word. For “bicycle/bicicleta” one of the judges suggested the use of a pseudoword so that the target could be reached. As for “iron/ferro”, one of the specialists proposed changing it to “vacation/férias” or “clay/barro” or “horseshoe/ferradura” or to the sentence “close the door/fecha a porta”.

The adaptation of “chewing gum/chiclete” to the target “chiclé”, which is more common in children's vocabulary and did not cause changes in phonemes or target positions. Therefore, both the elicitation of “chewing gum/chiclete” or “chewing gum/chiclé” are considered correct for completing the instrument. In contrast, the words “drone/zangão” and “zombie/zumbi” could not be changed, as there is no diversity of words with /z/ at the beginning of the word. The suggestion to use pseudowords was not accepted, as the purpose of the instrument is to search for quick naming without the frequent need for intervention by the applicator. Likewise, “iron/ferro” was not altered by the difficulty of visual representation of the suggestions “vacation/férias”, “clay/barro”, “horseshoe/ferradura” or “close the door/fecha a porta”. The lexical items “bucket/balde” and “snow/neve” were not changed due to the lack of justification and a new suggestion by the judge.

### Validity of response processes

**Figure 1:**
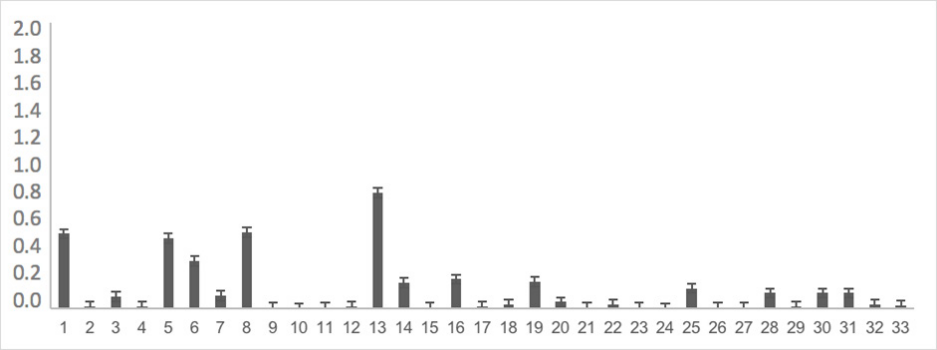
Average difficulty according to each item (items 1 to 33)

**Figure 2:**
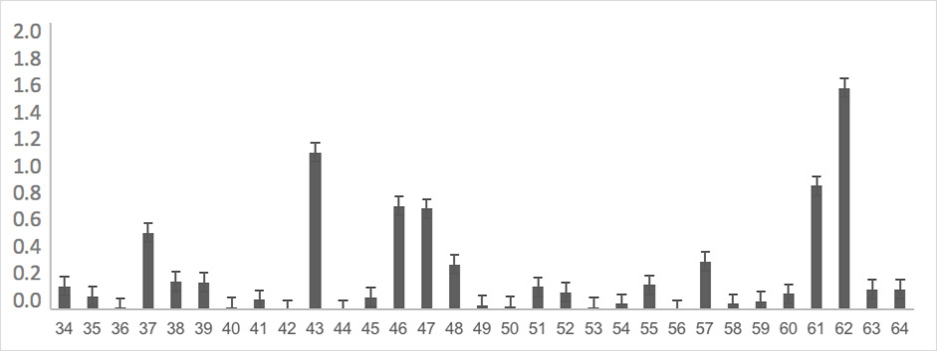
Average difficulty according to each item (items 34 to 64)

**Figure 3:**
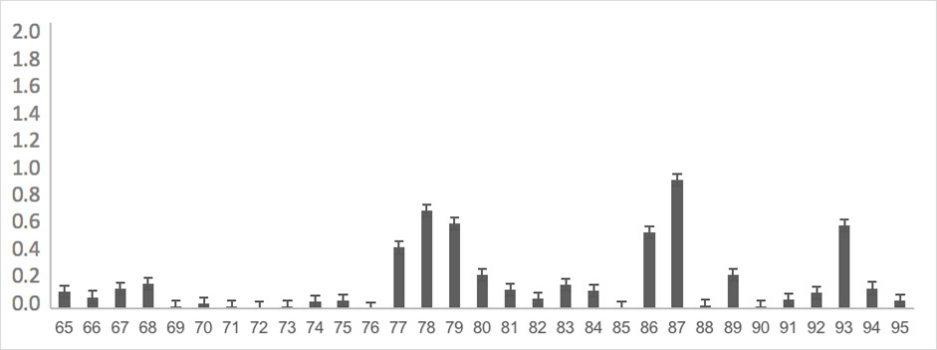
Average difficulty according to each item (items 65 to 95)

**Figure 4:**
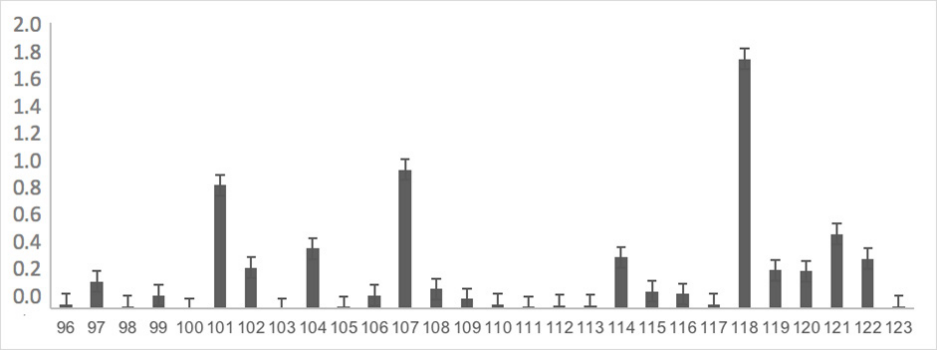
Average difficulty according to each item (items 96 to 123)

The instrument, already adapted, was completely applied to 176 children at school to obtain evidence of the validity of the response process. The most difficult items were: item 62 with 25% recognition in spontaneous naming, item 118 with 25% recognition, item 107 with 53% recognition, item 43 with 60% recognition, item 101 with 61% recognition and items 46, 87, and 47 with 69% recognition. As shown in Table 2, the rest of the words showed no difficulty in more than 70% of the analyzed sample. Figures 1, 2, 3, and 4 show the average difficulty per item, considering standard deviations.

**Table 2:** Item relation with the percentage of occurrence of response processes

Item	Response processes (%)				Item	Response processes (%)			
	Spontaneous nomination	Needed tips and/or close method	Required delayed imitation	Needed direct imitation		Spontaneous nomination	Needed tips and/or close method	Required delayed imitation	Needed direct imitation
1	62.5	23.3	13.6	0.6	63	87.5	10.8	1.7	0.0
2	98.9	1.1	0.0	0.0	64	88.1	9.7	2.3	0.0
3	94.9	2.3	2.3	0.6	65	92.0	4.5	2.8	0.6
4	98.3	1.1	0.0	0.6	66	94.3	3.4	2.3	0.0
5	53.4	43.2	2.8	0.6	67	87.5	11.4	1.1	0.0
6	77.8	10.8	11.4	0.0	68	84.7	13.1	2.3	0.0
7	94.3	2.3	3.4	0.0	69	98.9	0.6	0.6	0.0
8	59.7	25.6	13.6	1.1	70	96.6	1.1	1.1	1.1
9	99.4	0.6	0.0	0.0	71	97.7	1.7	0.0	0.0
10	100.0	0.0	0.0	0.0	72	99.4	0.6	0.0	0.0
11	99.4	0.6	0.0	0.0	73	98.3	1.7	0.0	0.0
12	98.9	0.6	0.6	0.0	74	97.7	0.0	2.3	0.0
13	42.6	33.5	23.9	0.0	75	96.0	2.3	1.7	0.0
14	86.9	7.4	4.5	1.1	76	100.0	0.0	0.0	0.0
15	99.4	0.6	0.0	0.0	77	61.9	33.5	4.5	0.0
16	81.8	15.9	2.3	0.0	78	46.0	38.6	14.8	0.0
17	98.9	1.1	0.0	0.0	79	42.0	54.0	2.3	1.7
18	96.6	2.8	0.0	0.6	80	77.3	21.0	1.1	0.6
19	81.8	16.5	1.1	0.6	81	89.8	6.3	3.4	0.6
20	95.5	4.5	0.0	0.0	82	95.5	2.3	2.3	0.0
21	99.4	0.6	0.0	0.0	83	91.5	1.1	6.8	0.6
22	98.3	1.1	0.0	0.6	84	93.2	1.1	5.7	0.0
23	99.4	0.6	0.0	0.0	85	99.4	0.6	0.0	0.0
24	100.0	0.0	0.0	0.0	86	64.8	17.6	17	0.6
25	86.4	13.1	0.6	0.0	87	43.2	25.6	29.5	1.7
26	99.4	0.6	0.0	0.0	88	98.9	0.0	1.1	0.0
27	99.4	0.6	0.0	0.0	89	77.8	17.6	2.8	1.7
28	91.5	6.3	2.3	0.0	90	98.3	1.7	0.0	0.0
29	98.9	1.1	0.0	0.0	91	95.5	2.8	1.7	0.0
30	89.8	9.7	0.6	0.0	92	90.9	6.8	2.3	0.0
31	91.5	6.8	1.1	0.6	93	42.0	56.3	0.6	1.1
32	97.2	2.8	0.0	0.0	94	91.5	2.8	5.7	0.0
33	98.3	1.1	0.6	0.0	95	96.6	1.1	2.3	0.0
34	83.5	15.9	0.0	0.6	96	98.9	0.0	0.6	0.6
35	90.9	9.1	0.0	0.0	97	89.8	1.7	8.5	0.0
36	98.9	1.1	0.0	0.0	98	98.9	0.6	0.6	0.0
37	61.4	23.9	14.8	0.0	99	93.8	3.4	2.8	0.0
38	80.7	18.2	0.6	0.0	100	100.0	0.0	0.0	0.0
39	88.1	5.1	6.8	0.0	101	54.0	6.8	38.1	1.1
40	98.9	0.6	0.6	0.0	102	81.8	9.1	8.0	1.1
41	95.5	2.3	2.3	0.0	103	100.0	0.0	0.0	0.0
42	100.0	0.0	0.0	0.0	104	67.6	21.0	10.2	1.1
43	31.8	28.4	38.1	1.7	105	98.3	1.1	0.0	0.6
44	100.0	0.0	0.0	0.0	106	93.8	3.4	2.8	0.0
45	91.5	8.5	0.0	0.0	107	50.0	2.8	46.0	0.6
46	59.1	9.7	31.3	0.0	108	90.9	4.5	4.5	0.0
47	61.9	7.4	29.0	1.7	109	92.6	5.7	0.6	1.1
48	82.4	4.5	12.5	0.6	110	98.3	0.6	1.1	0.0
49	97.2	1.7	0.6	0.6	111	99.4	0.0	0.6	0.0
50	97.7	2.3	0.0	0.0	112	97.7	2.3	0.0	0.0
51	88.6	6.8	4.5	0.0	113	98.3	1.1	0.6	0.0
52	88.6	10.8	0.6	0.0	114	65.3	33.5	1.1	0.0
53	98.3	1.7	0.0	0.0	115	88.6	10.2	0.0	1.1
54	96.0	2.8	0.6	0.6	116	92.6	4.5	2.8	0.0
55	86.9	9.1	4.0	0.0	117	98.3	0.6	1.1	0.0
56	100.0	0.0	0.0	0.0	118	1.1	24.4	70.5	4.0
57	77.8	11.4	10.2	0.6	119	84.7	4.0	11.4	0.0
58	97.2	1.7	1.1	0.0	120	81.3	13.6	2.8	2.3
59	96.6	1.1	2.3	0.0	121	65.3	17.6	15.3	1.7
60	89.8	7.4	1.7	1.1	122	80.7	4.0	15.3	0.0
61	15.3	82.4	1.7	0.6	123	71.6	0.0	0.6	27.8
62	22.7	2.3	72.7	2.3					

The form of intervention by the applicator, such as providing clues or using the delayed imitation feature, which stood out the most in each item, can be seen in Table 2. Items 62 and 118, “igloo/iglu” and “drone/zangão” stand out ”, which mostly required the use of delayed imitation and which, many times, were not uttered even with the use of this resource. The items that remained with recognition above 70% were the ones that least needed intervention from the applicators.

The instrument's internal consistency was calculated using Cronbach's Alpha and the 123 items had a consistency of 0.844. This result helps to infer that all the constituent elements are in agreement with each other^(18)^, which means that the interrelationship of the items supports the theoretically proposed structure.

## DISCUSSION

Considering the objectives of this study and the results shown, it was found that the IAF scores presented adequate indicators of content validity and response processes. Thus, the evaluated instrument can proceed to the next stages of validity and reliability.

It was possible to observe that those items highlighted by the expert judges, during the construction of content, were not necessarily the same items of difficulty of the target audience, during the analysis of the response processes. The exception was the word “iron/ferro”, one of the items identified as “inadequate” by the judges, which presented identification below 70% by the sample. In this sense, the validity based on the response processes gives indications of how the clinical application of the instrument will be. This happens in the same way that it allows the organization of manuals and additional guidelines for the applicators, in addition to the theoretical evaluation^(19)^.

Despite depending on the correct spontaneous naming of figures, the evaluation procedure demands the elicitation of target phonemes. This increases the possibility of the child's desired response. Accepting “tecla” for the item “keyboard/teclado”, for example, since the target phonemes remain identical. Likewise, “telha” for “roof/telhado”, “chiclé” for “chewing gum/chiclete” and “lixo” for “trash can/lixeira” are accepted.

The figures provide the necessary support to encourage natural speech since the collection must be as close as possible to the child's spontaneous oral language^(5)^. Seeking to meet the assessment demands, the applicator may have a sequence of strategies that encourage a speech closer to natural and estimate the subject's speech intelligibility. These strategies include the use of hints, the use of the close method, and the use of delayed imitation in the case of the IAF.

The use of hints is usually the most intuitive for the applicator. So that the child's engagement is not lost, dialogue with the child is maintained, favoring their commitment during the evaluation. The tips include small interventions to direct the subject's thinking, such as saying “It's the one you put in coffee” for item 2, “sugar/açúcar”; or “It is what it is, not its name” for item 87, “planet/planeta”, represented by the figure of the planet Saturn.

However, in various situations, there are more effective ways to obtain the desired response, such as using the close method resource. This resource, widely present in clinical practice, consists of using a phrase that leaves gaps for the target word ^(20)^. Using item 43, “explosion/explosão”, as an example: at a given moment, the applicator uses the tip “we call the fire department when that happens” and the child answers “fire/incêndio” to the target, needing more tips or even more delayed imitation to elicit correctly. At another time, the applicator uses the close method with the phrase “when it goes ‘BOOM’, we call it...” and promptly gets the answer “explosion/explosão”. This occurs because this method uses the skills of recurrent auditory and cognitive associations in the search for the answer, as recommended in speech therapy^(20)^.

When the child demonstrates greater difficulties in reaching the target, imitation resources can be used as a last alternative to guarantee to obtain a phoneme sample. Among these resources, however, priority is given to delayed imitation to the detriment of direct imitation. Direct imitation consists of the immediate repetition of the model provided by the instrument's applicator, while delayed imitation only allows repetition after a period of latency and distraction. Thus, in delayed imitation, the applicator provides the model and warns that he will return to the image in the sequence. Now it has become a mental image- the signifier, or the interiorized image that represents a content or object^(5)^.

It is reinforced that the instrument applicator must keep in mind the prioritization of spontaneity at the time of evaluation. The repetition provided by the imitation resource tends to make the speech truncated, in addition to masking the difficulty of sounds of the child with PD ^(5,21)^. In the context of phonological assessment, therefore, imitation should be used as a last resort for target elicitation.

A descriptive study that sought to analyze the validation procedures used in oral language assessment instruments^(22)^ resulted in the prevalence of studies with the presence of content validation, however, few carried out the reliability test using Cronbach's alpha. The present study demonstrated a high internal consistency estimate for the IAF (0.844), meaning that the responses obtained with the instrument are safe for evaluation. Another national instrument obtained a median of 0.816, also indicating a satisfactory consistency of the items that make up the instrument to assess BP phonemes^(12)^.

A systematic review of the evidence of validity in the development of instruments in speech therapy^(11)^ showed that no study found demonstrated results of all types concomitantly (based on the internal structure, the response process, the external criteria, and the content), which indicates the lack of improvement of studies in the field of speech therapy. From this, the search for evidence of content and response processes in the IAF is not enough to make it a gold standard for validity, according to Psychometrics^(9)^.

As it collects data from students from schools in the city of Porto Alegre/RS, the present study had limitations such as the use of reduced sample size and variability. Also in this sense, there was a failure in the application of the complete instrument in 3 children able to participate in the study, which may have influenced the results. It is important to emphasize that the IAF still has a long way to go for its validation, as it is necessary to establish construct and reliability standards with security. It also needs to carry out studies with representative population samples from the Brazilian territory.

The present study contributes to clinical practice based on scientific evidence in the field of language. By seeking evidence of content validity with expert judges, this study attests to the existence of an instrument close to the ideal quality proposed by the scientific community. On the other hand, when looking for evidence of the validity of the response process with non-specialist judges and children, this study confers attributes close to clinical practice and indicates which difficulties may arise during the evaluation.

## CONCLUSION

This study was able to demonstrate evidence of content validity and response process in the Phonological Assessment Instrument, IAF. Together, with this study, it was possible to adjust and improve the test items in a more judicious manner, benefiting clinical and scientific use.

## Footnotes

1 Phonological Disorder is the same as what ASHA calls Speech Sound Disorder and what DSM-5 calls Speech Disorder. However, the term Phonological Disorder was chosen, because both in ASHA and DSM-5, it is exposed in a general manner, encompassing both articulation and phonological disorders in the same denomination, and what is being approached specifically is PD.

2 Psychometric studies on the properties of the construct validity and reliability instrument were submitted in other articles due to the restriction of the number of pages, tables, and charts allowed by the journals, which would prevent including all the details of each step in a single article.
