# Supplementary material for: Phonological Assessment Instrument: evidence of content validity and response processes
Source: Codas. 2023 Oct 23;36(2):e20220324. doi: 10.1590/2317-1782/20232022324en (PMC10715633; doi:10.1590/2317-1782/20232022324en)
Supplement: Translation

# Translation: Instrumento de Avaliação Fonológica: evidências de validade de conteúdo e de processos de resposta

**DOI:** 10.1590/2317-1782/20232022324pt

**Authors:** Ana Carolina Sartori Bernardi, Camila Botura, Giovana Sopezack Alves, Letícia Pacheco Ribas

**Affiliations:** 1 Universidade de Ciências da Saúde de Porto Alegre - UFCSPA - Porto Alegre (RS), Brasil.; 2 Departamento de Fonoaudiologia, Universidade de Ciências da Saúde de Porto Alegre - UFCSPA - Porto Alegre (RS), Brasil.

**Keywords:** Estudo de Validação, Validade dos Testes, Fala, Criança, Testes de Linguagem, Transtorno Fonológico, Fonoaudiologia

## Abstract

**Objetivo:**

evidenciar validade de conteúdo e validade de processos de resposta de um instrumento destinado à avaliação fonológica de crianças.

**Método:**

validade realizada em duas etapas por dois diferentes grupos de juízes, grupo de especialistas e grupo de não-especialistas. O primeiro grupo composto por três juízes especialistas avaliaram os 123 itens lexicais após a elaboração do instrumento, julgando a aplicabilidade das figuras no contexto da avaliação infantil e sugerindo ajustes para compor o conteúdo. A partir das observações, o instrumento foi adequado e direcionado ao grupo de juízes não-especialistas que, por meio da aplicação do instrumento, tiveram suas respostas avaliadas conforme a facilidade ou dificuldade de elicitação dos itens do instrumento.

**Resultados:**

As avaliações obtiveram resultados de teor positivo para as validades de conteúdo e de processos de resposta.

**Conclusão:**

o estudo permitiu aprimorar os itens de teste de forma mais criteriosa, beneficiando o uso clínico e científico.

## INTRODUÇÃO

A linguagem se configura como uma habilidade inerentemente humana capaz de representar objetivamente o pensamento abstrato através de um sistema complexo de códigos compartilhados, que é a língua^(^ 1 ^)^. Funcionando a serviço da comunicação interpessoal, a linguagem é composta por domínios linguísticos que contemplam o uso, a forma e o conteúdo^(2)^. A fonologia compete ao estudo funcional dos fonemas, que são as unidades sonoras mínimas capazes de estabelecer a distinção entre vocábulos de uma mesma língua^(2)^.

Gradativamente, a linguagem é adquirida e desenvolvida através de uma hierarquia que inclui todos os domínios linguísticos. No caso da aquisição do domínio da fonologia, é preciso que a criança organize os diversos sons que compõem o sistema fonológico de sua língua materna para que este se estabilize. Estudos mostram que a aquisição típica da linguagem ocorre até os 5 anos de idade^(1,3)^, considerando a complexidade dos traços distintivos dos fonemas^(3)^.

Entretanto, quando não há o desenvolvimento adequado da fonologia, se observam erros de produção de fala, como é o caso do Transtorno Fonológico (TF)^^1^^ ^(3)^. Este é caracterizado^(4)^ em crianças acima de 4 anos de idade, que apresentam majoritariamente trocas consonantais na fala, tendo limiares auditivos dentro dos padrões de normalidade, não demonstrando alterações no léxico e na sintaxe com relação à linguagem expressiva, ausência de alterações neurológicas evidentes, assim como habilidades cognitivas normais e capacidade de compreensão intacta. Sendo assim, é necessária a realização de testes de audição, avaliação da linguagem e verificação da inteligência da criança, para garantir o correto diagnóstico do TF.

Para entender melhor tal quadro, além da realização de todas as avaliações que confirmam o diagnóstico, é necessário realizar uma efetiva avaliação fonológica, já que somente esta poderá descrever detalhadamente as trocas na fala do indivíduo^(^ 5 ^)^. É importante, portanto, que se tenha um método eficiente que avalie o inventário fonológico da criança, buscando um parâmetro de como está o seu desenvolvimento e a sua fala. A sistematização da avaliação permitirá a comparação acurada do sistema fonológico da criança com o da língua alvo, possibilitando a investigação minuciosa dos aspectos que compõem a fala^(6)^.

Todo esse processo diagnóstico deve ser composto por testes com interpretações válidas, confiáveis e precisas^(^ 7 ^)^ para que o diagnóstico seja o mais adequado possível. Para ser válido, o instrumento de avaliação deve reunir evidências de que mede realmente o que se propõe a mensurar. Para ser confiável, por outro lado, o teste precisa indicar se é reprodutível ao longo do tempo e se há controle dos erros de mensuração^(8,9)^.

Logo, para mensurar ao que se propõe, o instrumento deve passar por etapas de validade para recolher evidências, constituídas por validade de: conteúdo, processos de resposta e construto. A validade de conteúdo e a de processos de respostas são as que surgem durante a constituição do teste. A de conteúdo ocorre logo após a elaboração teórica de um instrumento e é realizada com o auxílio de um ou mais grupos de especialistas dispostos a julgar, independentemente, cada item que compõe o protótipo do teste. Na sequência, a de processo-resposta avalia parte do público-alvo; é importante perceber quais são as maiores dificuldades e facilidades encontradas pelos sujeitos durante a realização da avaliação para que o teste seja aprimorado. Já a de construto consiste na análise do instrumento no que tange à amostra representativa de um domínio^(8,10)^.

No Brasil, poucos são os instrumentos fonoaudiológicos que padronizam seus percursos metodológicos para elaboração de testes válidos e fidedignos^(11)^. A falta de validação dos protocolos de avaliação fonológica em Português Brasileiro (PB) pode prejudicar a segurança das evidências clínicas para traçar um diagnóstico preciso, uma conduta adequada e um planejamento de intervenção correto. Embora já existam testes disponíveis para auxiliar na avaliação e diagnóstico, os instrumentos mais utilizados^(12)^, como a Avaliação Fonológica da Criança (AFC)^(13)^ e o ABFW - Teste de Linguagem Infantil - Fonologia^(14)^ apresentam limitações e ainda não possuem indicadores psicométricos de validade e fidedignidade comprovados cientificamente^(11)^. Existem outros, como Instrumento de Avaliação Fonológica (INFONO)^(12)^ e o Instrumento de Avaliação de Fala para Análise Acústica (IAFAC)^(15)^, porém menos divulgados e ainda não disponíveis para o uso clínico. Portanto, é de extrema importância a realização de um estudo de validação para trazer avanços na área, buscando um padrão-ouro cientificamente comprovado para a avaliação do domínio fonológico em PB, auxiliando no processo diagnóstico, além de fornecer parâmetros para diversos estudos. Dito isso, o presente estudo objetiva analisar as evidências das etapas de conteúdo e de processos de resposta na validade do Instrumento de Avaliação Fonológica (IAF)^^2^^ , que já é utilizado por alguns fonoaudiólogos.

## MÉTODO

Este trabalho é aprovado pelo Comitê de Ética em Pesquisa (CEP) de uma universidade federal sob parecer número 5.045.533. A pesquisa corresponde a um estudo observacional, transversal controlado, descritivo e quantitativo, cujos dados foram utilizados para a validade de conteúdo e de processos de resposta do IAF.

O IAF é um *software* delineado para avaliar o sistema de sons da fala infantil de forma eficiente, minuciosa e otimizada. O instrumento foi elaborado com 123 palavras, pertencem ao vocabulário infantil, extraídas de histórias infantis populares, facilmente representadas em imagem ou foto e são do tipo substantivo, com uma imagem correspondente a cada item lexical. Os itens foram criteriosamente selecionados de forma que as palavras contemplassem todos os fonemas consonantais em todas as posições silábicas do PB, com cinco ocorrências de cada fonema e posição silábica, totalizando 235 possibilidades fonêmicas. A coleta da fala da criança deve ocorrer a partir da nomeação de cada uma das imagens, pela observação das ilustrações ou fotografias, que leva aproximadamente 10 minutos para a aplicação. O avaliador deve gravar o áudio da coleta de fala, e posteriormente, ouvir e observar as elicitações das crianças e registrar as informações no *software*. Tal processo leva em torno de 10 a 30 minutos, dependendo da prática e habilidade do avaliador. Após a inserção dos dados no instrumento, os resultados são gerados automaticamente e expressos em relatórios descritivos e quantitativos por: grau de severidade de fala, análise contrastiva, processos fonológicos e mudança de traços distintivos.

### Validade de conteúdo

Após a construção teórica do instrumento, foram convidados três juízes especialistas para realizar o julgamento dos 123 itens no protótipo. Participaram da pesquisa os juízes que assinaram Termo de Consentimento Livre e Esclarecido (TCLE) e que eram minimamente mestres em linguística com expertise em aquisição fonológica típica e atípica. Eles deveriam indicar o nível de adequação de cada item lexical para a proposta e escolher, entre duas opções, a imagem que melhor o representasse. Nessa etapa, deveriam responder à pergunta “A palavra-alvo é adequada para pertencer a um instrumento de avaliação de fala infantil?” de forma quantitativa, por meio de um formulário eletrônico, organizado em escala *Likert* ^(16)^ para cada item numerado de 1 a 4, conforme explicitado no Quadro 1.

**Quadro 1:** Escala Likert para classificação de conteúdo

N	Correspondência
1	Item não adequado a um instrumento de avaliação de fala infantil
2	Item necessita de revisão para ser adequado a um instrumento de avaliação de fala infantil.
3	Item adequado a um instrumento de avaliação de fala infantil, embora necessite de pequenas alterações.
4	Item absolutamente adequado a um instrumento de avaliação de fala infantil.

Para a abordagem qualitativa, os especialistas deveriam justificar suas escolhas com seus próprios critérios, registrados em comentários descritivos no mesmo formulário eletrônico. Para respostas 1 ou 2, os juízes foram orientados a sugerir ao menos uma nova palavra para substituição, considerando os mesmos aspectos elencados durante a elaboração do instrumento (isto é, contemplar todos os fonemas consonantais em todas as posições silábicas do PB). Para compreender o nível de correspondência intra e inter-juízes, foi realizado o cálculo de Índice de Validade de Conteúdo (IVC).

### Validade de processos de resposta

Após a devolutiva dos juízes especialistas, foi calculado o tamanho amostral necessário para determinar um coeficiente Kappa de 0.80, significativamente superior à 0.60, indicando boa concordância e com estimativa de 25% de prevalência de TF^(17)^. Para significância de 5% e poder de 80%, o resultado foi uma amostra mínima de 165 crianças.

O IAF foi aplicado em um grupo de estudantes, entre 5 anos e 8 anos e 11 meses, de uma escola pública da rede municipal da cidade de Porto Alegre. A amostra deste estudo é composta de dados de 176 crianças, com aquisição fonológica típica ou com TF, considerando que nenhuma possui alterações auditivas, neurológicas e/ou cognitivas, dificuldades escolares, histórico de atraso neuropsicomotor e/ou de intercorrências em gravidez ou parto, conferidas por meio de avaliação prévia e informações recolhidas em entrevista com os responsáveis. Todos os pais ou os responsáveis assinaram TCLE e Autorização para Uso de Áudio; e, no caso de crianças acima de 7 anos, essas também assinaram Termo de Assentimento.

Com base nas coletas de fala da amostra, um quarto juiz, independente e cegado, classificou as respostas dos sujeitos quanto ao nível de dificuldade observado em cada um dos itens lexicais. As respostas foram rotuladas conforme a necessidade de intervenção do aplicador, descrito no Quadro 2. A partir disso, calculou-se a consistência interna para o total dos itens do IAF pelo Alfa de Cronbach e pelo percentual de reconhecimento de cada um dos itens pela frequência de produção da palavra-alvo.

**Quadro 2:** Escala Likert para uso de processos de resposta

N	Correspondência
0	Nomeação espontânea.
1	Necessitou de dicas e/ou método close.
2	Necessitou de imitação retardada.
3	Necessitou de imitação direta.

## RESULTADOS

### Validade de conteúdo

A análise dos três juízes especialistas a respeito do protótipo do instrumento IAF indicou IVC de 0,98, o que representa um índice muito bom para a validade de conteúdo do teste. Para compor o instrumento foram escolhidas aquelas imagens em que pelo menos dois dos três juízes estavam de acordo.

Como pode ser observado na Tabela 1, a grande maioria dos itens lexicais apresentou adequação máxima, representada pelo código “4” na escala *Likert*, para pertencer a um instrumento de avaliação de fala infantil. Os juízes classificaram as palavras de acordo com seus próprios critérios, sendo eles: a palavra ser ou não frequente no universo infantil; a palavra ser ou não boa para verificação do alvo; e a palavra proporcionar nomeação espontânea adequada.

**Tabela 1:** Índice de validade de conteúdo (IVC) do instrumento de acordo com cada juiz

Juízes	IVC
A	1,0
B	0,98
C	0,96
Total	**0,98**
Polarização juízes	**0,95**

**Legenda:** IVC = Índice de validade de conteúdo

Os critérios descritos pelos juízes foram semelhantes, apesar de terem sido definidos de forma individual e independente em caixa de texto dissertativa no formulário utilizado, o que explica a porcentagem obtida no cálculo de IVC e a homogeneidade das respostas, em que 116 itens atingiram convergência máxima. No entanto, sete dos itens apresentaram divergências, foram eles: “balde”, “bicicleta”, “chiclete”, “ferro”, “neve”, “zangão” e “zumbi”. Nesses casos, apenas um dos três juízes considerou que as palavras-alvo não eram adequadas, o que ficou representado pelo IVC próprio de 0,75. Para a maioria dos itens não houve sugestão de nova palavra. Para “bicicleta” um dos juízes sugeriu uso de pseudopalavra para que o alvo fosse atingido. Já para “ferro”, um dos especialistas propôs a troca para “férias” ou “barro” ou “ferradura” ou para a oração “fecha a porta”.

Foi realizada a adaptação de “chiclete” para o alvo “chiclé”, que é mais comum no vocabulário infantil e não provocou alteração nos fonemas ou nas posições-alvo. Sendo assim, tanto a eliciação de “chiclete” ou de “chiclé” são consideradas corretas para o preenchimento do instrumento. Em contrapartida, as palavras “zangão” e “zumbi” não puderam ser alteradas, pois não há diversidade de vocábulos com /z/ no início da palavra. A sugestão para uso de pseudopalavras não foi acatada, pois o objetivo do instrumento é a busca pela nomeação rápida sem a necessidade frequente de intervenção do aplicador. Da mesma forma, “ferro” não foi alterado pela dificuldade de representação visual das sugestões “férias”, “barro”, “ferradura” ou “fecha a porta”. Os itens lexicais “balde” e “neve” não foram alterados pela falta de justificativa e nova sugestão por parte do juiz.

### Validade de processos de resposta

O instrumento, já adaptado, foi completamente aplicado em 176 crianças na escola para obtenção de evidências de validade de processo de resposta. Os itens de maior dificuldade foram: item 62 com 25% de reconhecimento em nomeação espontânea, item 118 com 25% de reconhecimento, item 107 com 53% de reconhecimento, item 43 com 60% de reconhecimento, item 101 com 61% de reconhecimento e os itens 46, 87 e 47 com 69% de reconhecimento. Conforme apresentado na Tabela 2, o restante das palavras demonstrou nenhuma dificuldade em mais de 70% da amostra analisada. As Figuras 1, 2, 3 e 4 demonstram a média de dificuldade por item, considerando os desvios-padrão.

**Tabela 2:** Relação de item com percentual de ocorrência dos processos de resposta

**Item**	**Processos de resposta (%)**				**Item**	**Processos de resposta (%)**			
	Nomeação espontânea	Necessita dicas e/ou método close	Necessita imitação retardada	Necessita imitação direta		Nomeação espontânea	Necessita dicas e/ou método close	Necessita imitação retardada	Necessita imitação direta
1	62,5	23,3	13,6	0,6	63	87,5	10,8	1,7	0,0
2	98,9	1,1	0,0	0,0	64	88,1	9,7	2,3	0,0
3	94,9	2,3	2,3	0,6	65	92,0	4,5	2,8	0,6
4	98,3	1,1	0,0	0,6	66	94,3	3,4	2,3	0,0
5	53,4	43,2	2,8	0,6	67	87,5	11,4	1,1	0,0
6	77,8	10,8	11,4	0,0	68	84,7	13,1	2,3	0,0
7	94,3	2,3	3,4	0,0	69	98,9	0,6	0,6	0,0
8	59,7	25,6	13,6	1,1	70	96,6	1,1	1,1	1,1
9	99,4	0,6	0,0	0,0	71	97,7	1,7	0,0	0,0
10	100,0	0,0	0,0	0,0	72	99,4	0,6	0,0	0,0
11	99,4	0,6	0,0	0,0	73	98,3	1,7	0,0	0,0
12	98,9	0,6	0,6	0,0	74	97,7	0,0	2,3	0,0
13	42,6	33,5	23,9	0,0	75	96,0	2,3	1,7	0,0
14	86,9	7,4	4,5	1,1	76	100,0	0,0	0,0	0,0
15	99,4	0,6	0,0	0,0	77	61,9	33,5	4,5	0,0
16	81,8	15,9	2,3	0,0	78	46,0	38,6	14,8	0,0
17	98,9	1,1	0,0	0,0	79	42,0	54,0	2,3	1,7
18	96,6	2,8	0,0	0,6	80	77,3	21,0	1,1	0,6
19	81,8	16,5	1,1	0,6	81	89,8	6,3	3,4	0,6
20	95,5	4,5	0,0	0,0	82	95,5	2,3	2,3	0,0
21	99,4	0,6	0,0	0,0	83	91,5	1,1	6,8	0,6
22	98,3	1,1	0,0	0,6	84	93,2	1,1	5,7	0,0
23	99,4	0,6	0,0	0,0	85	99,4	0,6	0,0	0,0
24	100,0	0,0	0,0	0,0	86	64,8	17,6	17	0,6
25	86,4	13,1	0,6	0,0	87	43,2	25,6	29,5	1,7
26	99,4	0,6	0,0	0,0	88	98,9	0,0	1,1	0,0
27	99,4	0,6	0,0	0,0	89	77,8	17,6	2,8	1,7
28	91,5	6,3	2,3	0,0	90	98,3	1,7	0,0	0,0
29	98,9	1,1	0,0	0,0	91	95,5	2,8	1,7	0,0
30	89,8	9,7	0,6	0,0	92	90,9	6,8	2,3	0,0
31	91,5	6,8	1,1	0,6	93	42,0	56,3	0,6	1,1
32	97,2	2,8	0,0	0,0	94	91,5	2,8	5,7	0,0
33	98,3	1,1	0,6	0,0	95	96,6	1,1	2,3	0,0
34	83,5	15,9	0,0	0,6	96	98,9	0,0	0,6	0,6
35	90,9	9,1	0,0	0,0	97	89,8	1,7	8,5	0,0
36	98,9	1,1	0,0	0,0	98	98,9	0,6	0,6	0,0
37	61,4	23,9	14,8	0,0	99	93,8	3,4	2,8	0,0
38	80,7	18,2	0,6	0,0	100	100,0	0,0	0,0	0,0
39	88,1	5,1	6,8	0,0	101	54,0	6,8	38,1	1,1
40	98,9	0,6	0,6	0,0	102	81,8	9,1	8,0	1,1
41	95,5	2,3	2,3	0,0	103	100,0	0,0	0,0	0,0
42	100,0	0,0	0,0	0,0	104	67,6	21,0	10,2	1,1
43	31,8	28,4	38,1	1,7	105	98,3	1,1	0,0	0,6
44	100,0	0,0	0,0	0,0	106	93,8	3,4	2,8	0,0
45	91,5	8,5	0,0	0,0	107	50,0	2,8	46,0	0,6
46	59,1	9,7	31,3	0,0	108	90,9	4,5	4,5	0,0
47	61,9	7,4	29,0	1,7	109	92,6	5,7	0,6	1,1
48	82,4	4,5	12,5	0,6	110	98,3	0,6	1,1	0,0
49	97,2	1,7	0,6	0,6	111	99,4	0,0	0,6	0,0
50	97,7	2,3	0,0	0,0	112	97,7	2,3	0,0	0,0
51	88,6	6,8	4,5	0,0	113	98,3	1,1	0,6	0,0
52	88,6	10,8	0,6	0,0	114	65,3	33,5	1,1	0,0
53	98,3	1,7	0,0	0,0	115	88,6	10,2	0,0	1,1
54	96,0	2,8	0,6	0,6	116	92,6	4,5	2,8	0,0
55	86,9	9,1	4,0	0,0	117	98,3	0,6	1,1	0,0
56	100,0	0,0	0,0	0,0	118	1,1	24,4	70,5	4,0
57	77,8	11,4	10,2	0,6	119	84,7	4,0	11,4	0,0
58	97,2	1,7	1,1	0,0	120	81,3	13,6	2,8	2,3
59	96,6	1,1	2,3	0,0	121	65,3	17,6	15,3	1,7
60	89,8	7,4	1,7	1,1	122	80,7	4,0	15,3	0,0
61	15,3	82,4	1,7	0,6	123	71,6	0,0	0,6	27,8
62	22,7	2,3	72,7	2,3					

**Figura 1:** Dificuldade média de acordo com cada item (itens 1 a 33)

**Figura 2:** Dificuldade média de acordo com cada item (itens 34 a 64)

**Figura 3:** Dificuldade média de acordo com cada item (itens 65 a 95)

**Figura 4:** Dificuldade média de acordo com cada item (itens 96 a 123)

A forma de intervenção do aplicador, como o fornecimento de pistas ou o uso do recurso de imitação retardada, que mais se destacou em cada item, pode ser conferida na Tabela 2. Destacam-se os itens 62 e 118, “iglu” e “zangão”, que necessitaram majoritariamente do uso de imitação retardada e que, muitas vezes, não foram proferidos mesmo com o uso desse recurso. Os itens que se mantiveram com reconhecimento acima de 70% foram os que menos necessitaram de intervenção dos aplicadores.

A consistência interna do instrumento foi calculada através do Alpha de Cronbach e os 123 itens apresentaram a consistência de 0,844. Esse resultado ajuda a inferir que todos os elementos constituintes estão de acordo entre si^(18)^, o que significa que a inter-relação dos itens permite suportar a estrutura teoricamente proposta.

## DISCUSSÃO

Considerando os objetivos deste estudo e os resultados exibidos, verificou-se que os escores do IAF apresentaram adequados indicadores de validade de conteúdo e de processos de resposta. Assim, o instrumento avaliado está apto a prosseguir para as próximas etapas de validade e de fidedignidade.

Foi possível observar que aqueles itens destacados pelos juízes especialistas, durante a construção de conteúdo, não necessariamente foram os mesmos itens de dificuldade do público-alvo, durante a análise dos processos de resposta. A exceção foi a palavra “ferro”, um dos itens apontados como “não adequado” pelos juízes, que apresentou identificação abaixo de 70% pela amostra. Nesse sentido, a validade com base nos processos de resposta dá indícios de como será a aplicação clínica do instrumento, da mesma forma que permite a organização de manuais e orientações adicionais aos aplicadores, para além da avaliação teórica^(19)^.

Apesar de depender da correta nomeação espontânea das figuras, o procedimento de avaliação demanda a elicitação dos fonemas-alvo. Isso amplia a possibilidade de resposta almejada da criança, aceitando-se “tecla” para o item “teclado”, por exemplo, já que os fonemas-alvos se mantêm idênticos. Da mesma forma, aceita-se “telha” para “telhado”, “chiclé” para “chiclete” e “lixo” para “lixeira”.

As figuras fornecem o apoio necessário para incentivar a fala natural, já que é importante que a coleta se aproxime ao máximo da linguagem oral espontânea da criança ^5^. Procurando alcançar as demandas de avaliação, o aplicador pode dispor de uma sequência de estratégias que incentivem uma fala mais próxima do natural e estimem a inteligibilidade de fala do avaliado. Essas estratégias incluem o uso de dicas, o uso do método close e a utilização do recurso de imitação retardada no caso do IAF.

O uso de dicas costuma ser o mais intuitivo para o aplicador. Para que o engajamento da criança não se perca, mantém-se o diálogo com a criança, favorecendo o empenho dela durante a avaliação. As dicas incluem pequenas intervenções para direcionar o pensamento do sujeito, tal como dizer “é aquele que se coloca no café” para o item 2, “açúcar”; ou “é o que ele é, não o nome dele” para o item 87, “planeta”, representado pela figura do planeta Saturno.

No entanto, em variadas situações há maneiras mais eficazes de se obter a resposta almejada, como a utilização do recurso de *método close*. Esse recurso, amplamente presente na prática clínica, consiste no emprego de uma frase que deixe lacunas para a palavra alvo^(20)^. Utilizando o item 43, “explosão”, como exemplo: em um determinado momento, o aplicador usa a dica “a gente chama os bombeiros quando isso acontece” e a criança responde “incêndio” para o alvo, necessitando de mais dicas ou ainda de imitação retardada para elicidar corretamente; em outro momento, o aplicador utiliza o método close com a frase “quando faz ‘BOOM’, a gente chama de...” e obtém prontamente a resposta “explosão”. Isso ocorre porque esse método utiliza as habilidades de associações auditivas e cognitivas recorrentes na busca pela resposta, assim como preconizado na terapia fonoaudiológica^(20)^.

Quando a criança demonstra maiores dificuldades para atingir o alvo, os recursos de imitação podem ser utilizados como última alternativa para garantia de obtenção de amostra de fonema. Dentre esses recursos, no entanto, prioriza-se a imitação retardada em detrimento da direta. A imitação direta consiste na repetição imediata do modelo fornecido pelo aplicador do instrumento, enquanto a imitação retardada só permite repetição após um período de latência e distração. Dessa forma, na imitação retardada, o aplicador fornece o modelo e avisa que retornará à imagem na sequência, tornando-se agora uma imagem mental, o significante ou a imagem interiorizada que representa um conteúdo ou objeto^(5)^.

Reforça-se que o aplicador do instrumento deve ter em mente a priorização da espontaneidade no momento de avaliação. A repetição proporcionada pelo recurso de imitação tende a tornar a fala truncada, além de mascarar os sons de dificuldade da criança com TF^(5,21)^. No contexto da avaliação fonológica, portanto, deve-se utilizar a imitação como último recurso para elicitação dos alvos.

Um estudo descritivo que buscou analisar os procedimentos de validação utilizados em instrumentos de avaliação de linguagem oral^(22)^ teve como resultado a prevalência de estudos com presença de validação de conteúdo, no entanto, poucos realizaram o teste de confiabilidade pelo alfa de Cronbach. O presente estudo demonstrou estimativa de consistência interna alta para o IAF (0,844), significando que as respostas obtidas com o instrumento são seguras para a avaliação. Outro instrumento nacional obteve mediana de 0,816 indicando, também, uma consistência satisfatória dos itens que compõem o instrumento para avaliar os fonemas do PB^(12)^.

Uma revisão sistemática sobre evidências de validade no desenvolvimento de instrumentos na Fonoaudiologia^(11)^ apresentou que nenhum estudo encontrado demonstrou resultados de todos os tipos concomitantemente (com base na estrutura interna, no processo de resposta, nos critérios externos e no conteúdo), o que indica a falta de aprimoramento dos trabalhos na área da Fonoaudiologia. A partir disso, tem-se que a busca por evidências de conteúdo e de processos de resposta no IAF não é suficiente para o tornar padrão-ouro para validade, conforme a Psicometria^(9)^.

Por coletar dados de alunos de escolas do município de Porto Alegre/RS, o estudo apresentado teve como limitações o uso de amostra reduzida em tamanho e variabilidade. Também nesse sentido, ocorreu falha na aplicação do instrumento completo em 3 crianças aptas a participar do estudo, o que pode ter influenciado os resultados. É importante ressaltar que o IAF ainda possui um longo caminho para sua validação, pois é preciso estabelecer os padrões de construto e fidedignidade com segurança, bem como realizar estudos com amostras populacionais e representativas do território brasileiro.

O presente trabalho contribui com a prática clínica baseada em evidências científicas no âmbito da linguagem. Ao buscar evidências de validade de conteúdo com juízes especialistas, este estudo atesta a existência de um instrumento próximo à qualidade ideal proposta pela comunidade científica. Por outro lado, ao buscar evidências de validade de processo de resposta com juízes não especialistas, as crianças, este estudo confere os atributos próximos à prática clínica e indica quais dificuldades podem surgir durante a avaliação.

## CONCLUSÃO

Este trabalho pôde demonstrar evidências de validade de conteúdo e de processo de resposta no Instrumento de Avaliação Fonológica, IAF. Conjuntamente, com este estudo, foi possível adequar e aprimorar os itens de teste de forma mais criteriosa, beneficiando o uso clínico e científico.

## Footnotes

1 O Transtorno Fonológico é o mesmo que a ASHA chama de Transtorno dos Sons da Fala e que o DSM-5 chama de Transtorno da Fala. Porém, preferiu-se optar por Transtorno Fonológico, pois tanto na ASHA quanto no DSM-5, ele é exposto de forma geral, englobando tanto os transtornos de articulação quanto fonológico na mesma denominação, e está se falando especificamente de TF.

2 Estudos psicométricos sobre as propriedades do instrumento de validade de construto e confiabilidade foram submetidos em outros artigos em função da restrição do número de páginas, tabelas e quadros permitidos pelas revistas, o que impediria de incluir todos os detalhes de cada etapa em um único artigo.
